# Uterine Mesenchymal Tumors: Updates on Pathology, Molecular Landscape, and Therapeutics

**DOI:** 10.3390/medicina60071085

**Published:** 2024-07-02

**Authors:** Amal A. Alodaini

**Affiliations:** Pathology Department, King Fahd University Hospital, College of Medicine, Imam Abdulrahman Bin Faisal University, P.O. Box 1982, Dammam 31441, Saudi Arabia; aaodaini@iau.edu.sa

**Keywords:** mesenchymal tumor, gynecology, pathology, molecular update

## Abstract

*Background:* Mesenchymal uterine tumors are a diverse group of neoplasms with varying biological potential. Many of these neoplasms can have overlapping morphologic similarities, which, in some instances, render their diagnosis and categorization thorough histomorphologic examination inconclusive. In the last decade, an exponential amount of molecular data aiming to more accurately characterize and, consequently, treat these tumors have accumulated. *Objective:* The goal of this narrative review is to provide a pathologic review, a genetic update, and to know the new therapeutic avenues of primary uterine mesenchymal neoplasms.

## 1. Introduction

**Figure 1:**
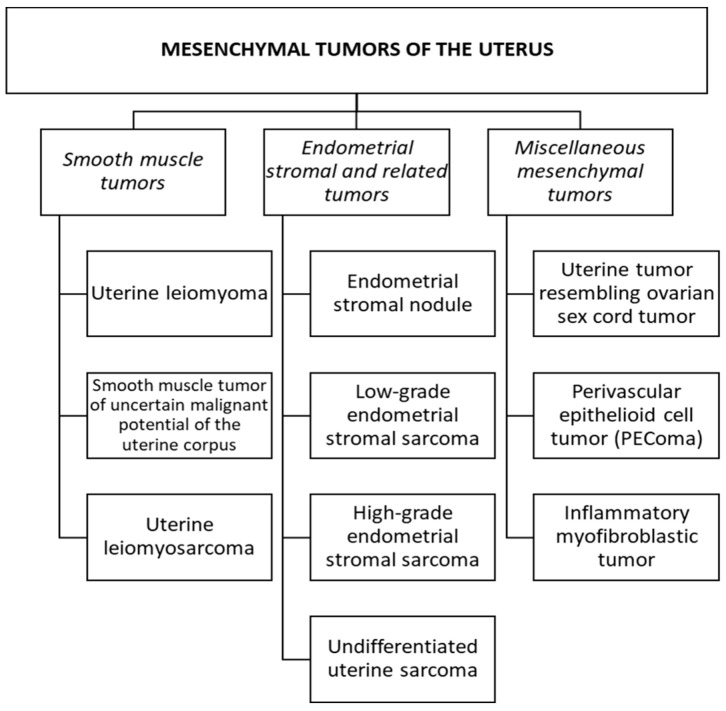
The World Health Organization’s 2020 classification of mesenchymal uterine tumors (adapted from WHO [1]).

Uterine mesenchymal tumors (UMTs) form a large, diverse group of non-epithelial tumors with varying malignant potentials. They constitute the second most common type of uterine corpus tumors after endometrial carcinomas, accounting for 8% of uterine corpus cancers [1,2]. Typically, UMTs are classified into unique subtypes based on their cellular origin (Figure 1, [1]), which can predict the distinct outcome and dictate their clinical management. Smooth muscle neoplasms are the most common uterine mesenchymal neoplasms, with leiomyosarcomas [ULMs] constituting 63% of uterine sarcomas, followed by endometrial stromal sarcoma [ESS, 21%], and other rarer entities that represent the remaining uterine mesenchymal tumors [2]. Occasionally, UMTs can pose a diagnostic challenge when they have overlapping morphologic and/or immunohistochemical features, especially with the increasing trends of limited sampling techniques, which can impede a precise tumor classification. In recent years, UMTs have been extensively examined using advanced molecular techniques, which have provided insights into their genetic landscape, identifying specific “driver” genes and uncovering new entities with distinct biological behaviors (Table 1). The interest in identifying the genetic signature of UMTs is not only of diagnostic value but also serves as a theragnostic tool that can guide treatments based on the targeting of underlying genetic drivers that, subsequently, broaden patients’ treatment options. This article reviews the latest literature on established and emerging UMTs, including their pathological features and molecular landscape, as well as their novel therapeutics.

## 2. Uterine Leiomyosarcomas [ULMs]

ULMs are malignant mesenchymal tumors of smooth muscle derivation. Although ULMs account for ~40–50% of uterine sarcomas, they represent ~1–2% of all uterine cancers. They commonly affect patients over the age of 50 years. Patients present with vaginal hemorrhage, a pelvic mass, or abdominal/pelvic pain. Regional spreading may cause gastrointestinal or urinary tract manifestations. In rare cases, the presenting symptoms can be due to the tumor rupture with subsequent hemoperitoneum or pulmonary metastases, resulting in dyspnea [1].

### 2.1. Pathological Features

**Figure 2:**
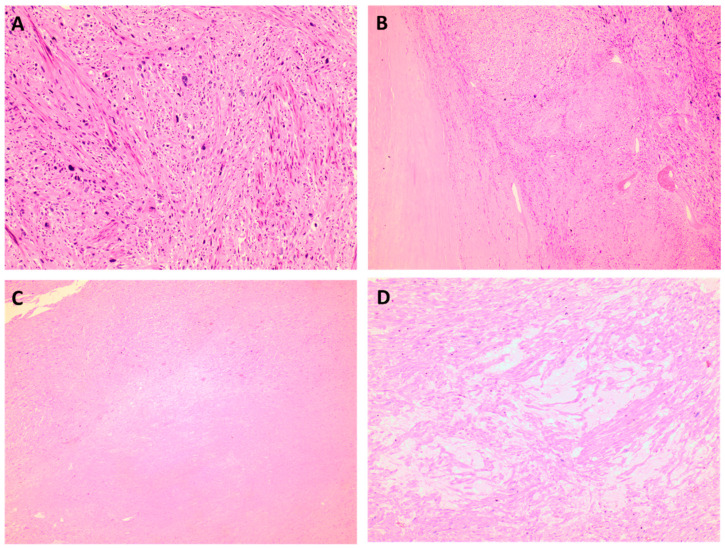
(**A**) Spindle-cell (conventional) tumors consist of fusocellular cells with eosinophilic cytoplasm arranged in long, interlacing fascicles with readily visible nuclear pleomorphism [H&E, 10×]. (**B**,**C**) Tumor cell necrosis characterized by a geographical distribution and sudden shift from viable to non-viable tumor cells [H&E, 20× and 10×]. (**D**) Myxoid stroma in myxoid leiomyosarcomas [H&E, 10×].

There are three recognized morphological subtypes: spindle-cell, epithelioid, and myxoid. Spindle-cell (conventional) tumors consist of fusiform cells with an eosinophilic cytoplasm grouped in long, interlacing fascicles (Figure 2). Although nuclear pleomorphism is readily visible, a proportion of these tumors have homogeneous cytological characteristics and may seem benign at low magnifications. Atypical mitoses are present, and the mitotic count is usually high (4 mitoses/mm^2^, equal to 10 mitoses/10 HPF of 0.55 mm in diameter). Tumor necrosis, characterized by a sudden shift from viable to non-viable tumor cells, is present in one-third of cases. Epithelioid leiomyosarcomas comprise round polygonal cells grouped into nested, corded, or diffuse patterns that constitute more than 50% of the tumor cells. Finally, myxoid leiomyosarcomas are paucicellular with abundant myxoid stroma (Figure 2). They may exhibit a hazy fascicular or nodular development. Extensive sampling may be required to identify the areas of malignancy [32]. Any cytological atypia, tumor cell necrosis, or more than 1 mitosis/10 HPF (in a 0.55 mm in diameter) is diagnostic of myxoid leiomyosarcoma. The tumors frequently have infiltrative borders, and vascular space invasion occurs in 20% of cases. Tumor cells express desmin, h-caldesmon, and SMA; however, their expression may be weak and/or patchy if the tumor is poorly differentiated or myxoid. CD10, EMA, and cytokeratin positivity are variable, but these are more common in epithelioid subtypes [33,34]. Estrogen and progesterone receptors (ER and PR, respectively) are frequently expressed in spindle-cell leiomyosarcomas. P16 and/or p53 overexpression is prevalent.

### 2.2. Molecular Landscape and Novel Therapeutic Approach

ULMs lack a defining genomic alteration with low tumor mutational burden [TMB] and complex genetic landscape resulting in complex somatic mutations and chromosomal numerical and structural aberrations. The commonly mutated genes include TP53 (~30%), RB1, PTEN, and ATRX (~25%), which are involved in tumor suppression and DNA damage response [3,4]. Progesterone receptor (PR, 11q22) gene rearrangement, mostly with the NR4A3 gene, was recently detected in epithelioid ULMs with rhabdoid and spindle cell features [5,6]. PGR fusion-positive epithelioid ULMs appear to have indolent behavior [5]. More than 25% of myxoid ULMs harbor pleomorphic adenoma gene 1 (PLAG1, 8q12) fusions. The fusion partners include the TRPS1 and RAD51B genes [35]. The PLAG1 gene encodes a protein that controls the cell cycle and inhibits GATA-mediated transcription. There are similar rearrangements present in pleomorphic adenoma, carcinoma ex-pleomorphic adenoma, and skin and soft tissue myoepithelioma [6,7].

Patients with ULMs are treated with radical hysterectomy with or without adnexectomy and/or lymph nodes. Unresectable or metastatic ULM cases are treated with doxorubicin-based chemotherapy or gemcitabine in combination with docetaxel as a first-line regimen [35]. However, in general, ULMs show a limited response to chemotherapy [1]. New treatment approaches are discussed below.

**Immunotherapy.** The status of the ULM immune microenvironment was variable among different studies [36,37,38,39], and PD-1 checkpoint blockade was ineffective. Instead, ULMs were identified as being enriched with tumor-associated macrophages [TAMs], which were linked to worse survival outcomes. TAMs contribute to cancer progression through angiogenesis and tumor immune evasion. Tumor cells expressing colony-stimulating factor-1 [CSF-1] bind to the CSF-1 receptors [CSF-1Rs] on macrophages in the tumor microenvironment to facilitate tumor invasion. Currently, a CSF-1R inhibitor [DCC-3014] is the subject of experimentation in combination with doxorubicin and a CD40 agonist to inhibit the CSF-1/CSF-1R axis [NCT 0424238] [40]. Another approach for modulating the phagocytic action of macrophages in the tumor microenvironment is through inhibition of the binding of CD47, a transmembrane protein widely expressed by tumor cells, to the signal regulatory protein alpha [SIRP-alpha] on the macrophage surfaces through anti-CD47 monoclonal antibodies [TTI-621, NCT049996004] [35]. Another proposed immune-therapy-based method is increasing the tumor antigenicity burden through the utilization of poly (ADP-ribose) polymerase [PARP] inhibitors to induce DNA damage in ULMs [NCT 04624178] [36].

**Targeting DNA repair pathway.** Several studies have shown that ULMs harbor frequent defects in the homologous recombination [HR] DNA repair pathway [41,42,43,44]. As such, using PARP inhibitors can potentiate the death of tumor cells through inhibiting DNA repair and inducing progressive genomic instability [36]. Case reports have described the efficacy of PARP inhibitor monotherapy for patients with BRCA2 deletion [36]. A study tested the olaparib/temozolomide combination in ULM patients and showed progression-free survival of 6.9 months and a 12-month median response duration [45]. The correlative genetic analysis in these patients demonstrated an alteration in HR-DNA genes, including PALB2 and RAD51B [45]. A novel therapeutic agent, ATX-101, is being tested in a phase II clinical trial as a monotherapy for patients with ULMs [NCT05116683] [36].

**Targeting receptor tyrosine kinases.** No recurrent oncogenic tyrosine kinase receptor (TKR) alterations have been identified in ULMs until now, which limits the utilization of highly effective and selective TKR inhibitors identified in other soft tissue sarcomas. On rare occasions, ALK, EGFR, and NTRK alterations can be identified in ULMs in which they can be targeted by TKR inhibitors. Broadly acting TKR inhibitors have been tested in ULMs, with minimal effects on the objective response rate and median progression-free survival [36]. Multi-receptor tyrosine kinase inhibitors [TKIs] targeting endothelial growth factor receptor 2/3, platelet drive growth factor A/B, fibroblast growth factor receptor 1–4, and RETm DDR1 showed improved progression-free survival [NCT03016819] [36].

## 3. Endometrial Stromal Tumors [ESTs]

ESTs are uterine neoplasms that originate from the functional endometrial stromal cells, and account for less than 1% of all uterine cancers [1]. The 2020 WHO classification divides ESTs into different subtypes with distinct cytogenetic features (Figure 1 and Table 1) [1]. Unlike uterine smooth muscle tumors, which are mostly benign, ESTs are almost always malignant. Low-grade endometrial stromal sarcoma (LG-ESS) and high-grade endometrial stromal sarcoma (HG-ESS) affect women between the ages of 40 and 55 years. The most common symptoms are abnormal uterine bleeding and pelvic discomfort. Other symptoms include dysmenorrhea, abdominal discomfort, and increased abdominal girth [1]. Approximately 70% of patients with HG-ESS have peritoneal, lung, intra-abdominal lymph nodes, and bone metastasis at presentation. The gynecological examination commonly reveals an irregular expanded uterus or a polyp-like tumor emerging through the cervical os.

### 3.1. Pathological Features of ESTs

**Figure 3:**
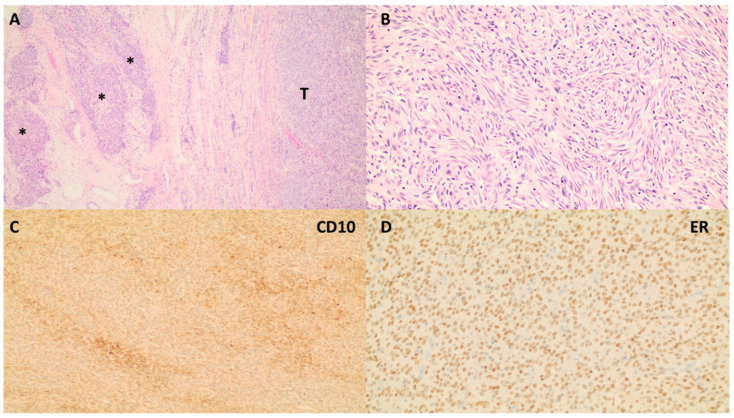
A low-grade endometrial stromal sarcoma. (**A**) The infiltrative tumor [T] growth pattern with irregular tongue-like extension [*] into the myometrium [H&E, 4×]. (**B**) Monotonous round to spindle cell proliferation in vague whirling pattern around spiral-like arterioles [H&E, 4×]. (**C**) Tumor cells are diffusely positive for endometrial stromal marker CD10 [4×]. (**D**) Tumor nuclei are strongly positive for estrogen receptor [ER, 10×].

Histologically, LG-ESS forms myometrial coalescent masses of proliferative-phase endometrial stromal cells that permeate the myometrium (Figure 3), with or without lymphovascular invasion [1]. The tumor cells in LG-ESS show minimal atypia and a delicate arteriolar network. Mitotic activity is usually inconspicuous; however, in rare cases, the activity can be as high as 10 mitotic figures/10 HPF. Through the use of immunohistochemistry, tumor cells have been shown to express CD10, ER, PR, and androgen receptor (AR) in 71–93%, 71–95%, and 59–73% of instances, respectively [46]. SMA and, less frequently, desmin can also be detected. HG-ESS exhibits tongue-like myometrium infiltration of monotonous stromal cells with high-grade atypical nuclei exceeding the level acceptable for LG-ESS. Bleeding and necrosis are frequently present with elevated mitotic activity [>10 mitotic figures/10 HPF]. Certain morphologic features are associated with certain genetic aberrations. Round eosinophilic cells with high-grade nuclei and uneven nuclear outlines, vesicular chromatin, and variably distinguishable nucleoli are characteristic of tumors with the YWHAE-NUTM2A/B fusion [1]. These tumors are positive for cyclin D1, BCOR, KIT, CD56, and CD99 [1]. ZC3H7B-BCOR high-grade endometrial stromal sarcomas can display striking myxoid changes, as well as spindle cells forming fascicles, and, thus, are frequently misdiagnosed as myxoid leiomyosarcomas. These diffusely express cyclin D1 and CD10, while BCOR, ER, PR, SMA, and h-caldesmon are variably expressed, and are negative for desmin using immunohistochemistry [1]. BCOR internal tandem duplication (ITD) HG-ESS have different immunohistochemistry expression profiles that are positive for cyclin D1, BCOR, and desmin, negative for ER, PR, SMA, and desmin, and variable for CD10 [1]. HG-ESS may show pan-tyrosine receptor kinase [pan-TRK] staining, but this is unrelated to NTRK rearrangement [47].

### 3.2. Molecular Landscape and Novel Therapeutic Approach

Several genetic mutations have been identified in low-grade and high-grade ESS. Nearly all LG-ESS harbor a recurrent chromosomal translocation of the JAZF1 (7p15) gene with one of the polycomb genes including SUZ1, PHF1, and EPC [1,45]. HG-ESS has different subsets of recurrent genetic rearrangements that include YWHAE-NUTM2A/B fusions [9,10,11,12,13,48], ZC3H7B-BCOR fusions [14,15,49,50], or BCOR ITD [16,17,35]. EPC1-BCOR, JAZF1-BCORL1, and BRD8-PHF1 fusions are rare genetic events [1]. Both LG-ESS and HG-ESS have a low mutational burden [36].

Total abdominal hysterectomy with bilateral salpingo-oophorectomy (TAH and BSO) are the standard initial treatment of patients with LG-ESS and HG-ESS. Hormone blockade and sarcoma-based chemotherapy are also used in advanced LG-ESS and HG-ESS, respectively, with a variable response. Both LG-ESS and HG-ESS have a low mutational burden which may predict a weak response to immune checkpoint inhibition [36]. However, ESS genomic translocations may still result in the development of potent tumor antigens that may be immunogenic. As such, Nivolumab is the subject of an ongoing investigation [NCT03241745] [36], and further research on the tumor immune milieu in ESS is required to guide these immunotherapy efforts. Studies have shown that proteins involved in the Wnt signaling pathway might be disrupted as downstream targets through various mechanisms in JAZF1-SUZ12, JAZF1-PHF1, and EPC1-PHF1-rearranged LG-ESS, resulting in Wnt activation [8,40,51]. Accordingly, the increased nuclear β-catenin in LG-ESS strongly justifies investigation of the Wnt pathway inhibitors as a therapeutic option. A gene expression profile study of HG-ESS demonstrated an increased expression of NTRK3, FGFR3, RET, GLI1, and PTCH1 and a low expression of ESR1 [52]. This was also noticed on a protein level, where HG-ESS were positive for pan-TRK under immunohistochemistry [36]. As a result, the role for TKI therapy, including pazopanib and imatinib, in HG-ESS patients has been tested and showed variable responses [40,51,53]. Due to the oncogenic effects of HG-ESS gene rearrangement on the cell-cycle checkpoints and the downstream activation of cyclin D1 [54,55] or MDM2 amplification [55], CDK4/6 inhibitors and MDM2 inhibitors are another potential therapeutic option for HG-ESS that are currently being investigated in preclinical [54] and clinical trials [36].

## 4. Perivascular Epithelioid Cell Neoplasms

These rare tumors are derived from the neural crest stem cells that are capable of myoid and melanocytic differentiation. Perivascular epithelioid cell tumor (PEComa) and lymphangioleiomyomatosis (LAM) are examples of perivascular epithelioid cell neoplasms that arise in the uterine corpus. While PEComa affects a wide age range of patients (28–75 years), LAM tends to occur in premenopausal women (mean age: 37 years). PEComas and LAMs are typically discovered incidentally when treated as a uterine fibroid and symptoms, which may include abnormal uterine/postmenopausal bleeding, abdominal pain, mass formation, extrauterine involvement, or rarely hemoperitoneum (especially in pregnant ladies), rarely occur [1]. The prognosis for a PEComa is variable [see below]. The most common recurrence sites are the lung, liver, abdomen, vagina, and lymph nodes.

### 4.1. Pathologic Features

**Figure 4:**
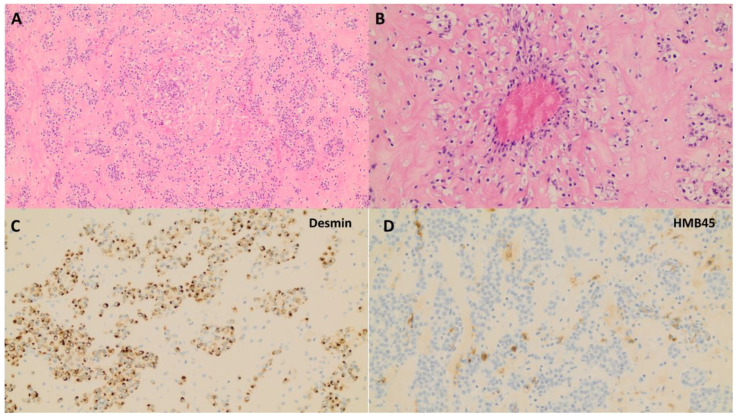
Perivascular epithelioid cell tumor (PEComa). (**A**) Epithelioid and/or spindled cells arranged in discohesive nests with delicate thin-walled vessels [H&E, 4×]; (**B**) Radial/perivascular distribution of tumor cells with clear-to-eosinophilic granular cytoplasm and stromal hyalinization are characteristic features and variably present [H&E, 20×]; (**C**) Tumor cells are positive for smooth muscle marker desmin [20×]; (**D**) Tumor cells show scattered positivity for melanocytic marker HMB45 [20×].

PEComas can have an expansive, permeative (tongue-like), or infiltrative growth pattern [56,57,58]. Conventional PEComas are composed of epithelioid and/or spindled cells with a clear-to-eosinophilic granular cytoplasm (Figure 4). The epithelioid cells are arranged in discohesive nests with delicate thin-walled vessels, whereas the spindled cells often form fascicles. The radial/perivascular distribution of tumor cells, multi-nucleated cells, cells with lipid-rich or rhabdoid cytoplasm, and stromal hyalinization are characteristic features that variably present. TFE3 translocation-associated PEComas have uniform epithelioid cells with either purely clear or clear-to-eosinophilic cytoplasm. Variable cytological atypia and mitotic activity, as well as melanin pigment (focal or diffuse), can be present. Conventional PEComas express HMB45 (most sensitive), melan-A, and smooth muscle markers (SMA, desmin, and h-caldesmon), with a variable intensity and extent. Cathepsin-K is diffusely and strongly positive; ER and PR are frequently positive; TFE3 is positive in tumors with diffuse epithelioid and clear morphology; and S100, CD117, and less commonly CD10, may rarely be positive in PEComas. PAX8, cytokeratin, CD34, SOX10, and inhibin are negative. PEComas tend to behave aggressively if three or more of the following morphologic features are met: a tumor size greater than 5 cm, high-level cytologic atypia, mitoses greater than 1/50 HPF, necrosis, and lymphovascular invasion.

### 4.2. Molecular Landscape and Novel Therapeutic Approach

Inactivating mutations of the tuberous sclerosis complex [TSC1/TSC2] genes lead to the upregulation of mTOR signaling [59]. Most cases are sporadic; however, ~10% of cases are associated with tuberous sclerosis syndrome [1]. A subset of tumors has TFE3 (9Xp11.2), RAD51B, or HTR4-ST3GAL1 fusions [18,19,58,60]. TSC mutations and TFE3 fusions are mutually exclusive [19,61]. Limited data indicate that tumors with RAD51B fusions may be more aggressive [19,58].

PEComas are traditionally treated with TAH ± BSO. In malignant PEComas, radiation and/or chemotherapy are added. A retrospective series has demonstrated that uterine PEComas are responsive to hormonal therapy [36] and that mTORC1 inhibitors [e.g., sirolimus, everolimus, and temsirolimus] can be considered because of the activation of the mTOR signaling pathway [36].

## 5. Inflammatory Myofibroblastic Tumor (IMT)

An IMT is a myofibroblastic-derived mesenchymal neoplasm enriched with lymphoplasmacytic inflammatory cells. IMTs have been described in multiple anatomical sites [1,20]; however, there are less than 100 uterine IMT cases reported [1]. This tumor occurs across a wide age range, but typically affects women in early to mid-adulthood [20]. The presenting symptoms include abdominal mass, pain, abnormal vaginal bleeding, and constitutional manifestations such as fatigue and/or fever [1,20].

### 5.1. Pathologic Features

Most of the cases are submucosal and limited to the uterine corpus, with rare examples of extrauterine extension. These tumors have infiltrative borders with a soft white-to-tan cut surface, variable foci of hemorrhage, and myxoid changes. Histologically, IMTs are composed of intersecting fascicles of spindle cells with pale eosinophilic cytoplasm and round to ovoid nuclei with variable atypia. The stroma can range from myxoid to densely collagenous and contain dense lymphoplasmacytic inflammatory infiltrate with different proportions of other inflammatory cells, including multi-nucleated giant cells. Lymphovascular invasion and necrosis can be present. The tumor is variably immunoreactive for desmin, SMA, h-caldesmon, and CD10 [1]. Contrary to IMTs present at other anatomic sites, 77% to 100% of uterine IMTs express ALK under immunohistochemistry in variable patterns; however, the combined granular and cytoplasmic expression is the most common pattern [21].

### 5.2. Molecular Landscape and Novel Therapeutic Approach

IMTs frequently harbor ALK (2p23) gene rearrangements with a variety of fusion partners, including IGFBP5, THBS1, FN1, DES, TIMP3, DCTN1, SEC. 31, TPM3, and PPP1CB [1,21]. TIMP3 and THBS1 are the most common partners in placenta-based IMTs [62].

IMTs are tumors of an uncertain behavior; the majority of uterine IMTs are benign and limited to the uterus, with a small percentage of tumors that can either spread outside of the uterus or recur [62,63,64,65,66]. For uterine-confined cancers, there are inadequate data to predict the prognosis. An aggressive course of behavior has been linked to necrosis, a tumor size greater than 7 cm, moderate-to-severe atypia, strong mitotic activity, and lymphovascular invasion [62,63]. TKIs can be used as a targeted therapy in IMTs with ALK rearrangements [67,68].

## 6. Uterine Tumors Resembling Ovarian Sex Cord Tumor (UTROSCT)

UTROSCTs are extremely rare mesenchymal neoplasms representing less than 1% of all uterine neoplasms that mostly affect the uterine corpus and rarely the cervix. UTROSCTs predominate in middle-aged women [mean age of 50 years old], in whom the tumor can be discovered incidentally or present with abnormal vaginal bleeding or pelvic pain. The tumor histogenesis is unclear; however, it has been hypothesized that these tumors arise from pluripotent mesenchymal, endometrial stromal, or displaced ovarian sex cord cells [20]. Most cases follow a benign course, and complete excision, or simple hysterectomy, would be curative [21]. However, a recent series reported that 23.5% of patients developed metastases and that 8.8% of patients died of their tumor [69]. As a result, it has been suggested that these tumors are more appropriately considered to be of uncertain malignant potential [21].

### 6.1. Pathologic Features

**Figure 5:**
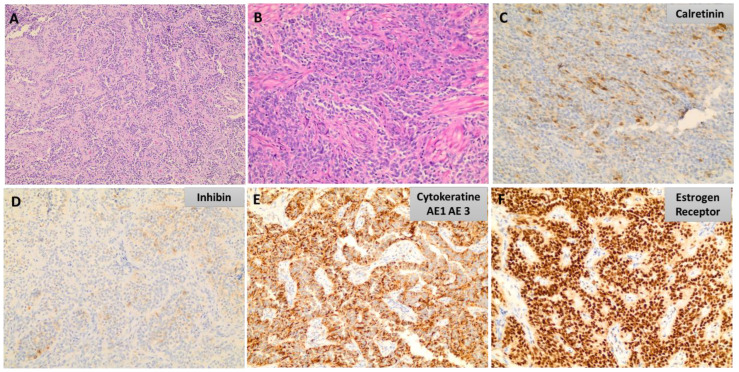
Uterine tumors resembling ovarian sex cord tumor (UTROSCT). (**A**,**B**) Epithelial-like cells arranged into sheet and retiform-like formations resembling sex cord-stromal tumors of the ovary. The nuclei are ovoid with minimum cytological atypia [H&E, 10× and 20×]. (**C**–**F**) Polyphenotypic immunohistochemical profile with varied positivity for sex cord markers (calretinin, inhibin), epithelial markers, and estrogen receptors [10× and 20×].

UTROSCTs are generally well circumscribed; however, some have an uneven margin with myometrial entrapment. They are composed of epithelial-like cells with morphological architectural patterns resembling the sex cord-stromal tumors of the ovary without recognizable components of endometrial stroma (Figure 5). The tumor cells are arranged into nests, trabeculae, or tubules, and can assume a retiform appearance [70]. The cytoplasm ranges from scant to abundant with pale-to-eosinophilic rhabdoid-like appearance. The nuclei are ovoid with minimum cytological atypia and modest mitotic activity and vascular invasion can occur. Evidently malignant tumors may exhibit conspicuous cytological atypia and brisk mitotic activity. These tumors exhibit a polyphenotypic immunohistochemical profile, with a varied positivity for sex cord markers (inhibin, calretinin, WT1, CD56, CD99, SF1, FOXL2, and melan-A), epithelial markers, ERs, PR smooth muscle markers (actin, desmin, and h-caldesmon), and CD10 [1].

### 6.2. Molecular Landscape and Novel Therapeutic Approaches

Rearrangements involving either ESR1 or GREB1 have been reported with a variety of partners, including NCOA1, NCOA2, NCOA3, CTNNB1, NR4A3, and SS18 [22,25]. These tumors do not harbor the molecular alterations seen in low-grade endometrial stromal sarcoma (JAZF1-SUZ12), adult granulosa cell tumor (FOXL2 alterations), or Sertoli–Leydig cell tumor (DICER1) alterations [26,27,69], and possess a poly phenotypic immunophenotype.

## 7. Müllerian Adenosarcoma

Müllerian adenosarcoma is a biphasic neoplasm composed of a benign epithelial component and a malignant stromal component. It is presumed to arise from the neoplastic transformation of a Müllerian mesenchymal cell that stimulates reactive growth in the benign glands [1] and can arise in uterine or from extrauterine locations from endometriosis [1]. Although it can affect a wide age range, most patients are typically postmenopausal, with some cases linked to tamoxifen use [1]. Patients complain of abnormal uterine hemorrhage, an enlarged uterus, or tissue protruding through the cervical os, with a history of recurring polyps being usual. In the absence of adverse prognostic features, the prognosis is generally favorable [see below] [2].

### 7.1. Pathologic Features

Grossly small adenosarcomas resemble typical endometrial polyps and are usually solitary, exophytic, and polypoid, filling the endometrial cavity and protruding through the cervical os. Adenosarcomas may contain cysts, resulting in a “spongy”, cut surface. Necrosis and hemorrhage are prevalent in large adenosarcomas, especially in the presence of sarcomatous overgrowth [1]. Histologically, the tumor has phyllodiform cleft-like or dilated glands (rigid cysts) lined with a benign endometrial epithelium surrounded by neoplastic stroma, which is generally hypercellular relative to the surrounding benign tissue and is referred to as “Peri-glandular cuffing” [2]. The neoplastic stroma is similar to endometrial-type stroma, although it can be fibroblastic, with plump cells and varied mitotic activity [1]. Cytologic atypia in the stroma is typically minimal. High-grade malignant stromas are uncommon but can be widespread and characterized by marked nuclear pleomorphism and more than 10 mitoses/10 HPFs [2]. Sarcoma overgrowth is described when more than 25% of the tumor is composed of solely neoplastic stroma, frequently of a high grade (often with rhabdomyoblastic differentiation), but in rare circumstances it can be a low grade. Immunohistochemically, the tumors are often positive for CD10, ER, and PR, although these are often negative in sarcomatous overgrowth [1]. The p53 expression by immunohistochemistry is highly correlated with the mutation status (see below) [2]. Desmin, myogenin, and MyoD1 help to identify a rhabdomyosarcoma component. The histologic criteria indicating a poor prognosis are sarcomatous overgrowth, high-grade components, deep myometrial invasion, and lymphovascular invasion [2].

### 7.2. Molecular Landscape and Novel Therapeutic Approach

This neoplasm lacks the genomic aberrations and specific chromosomal rearrangements found in other gynecological sarcomas. Low-grade adenosarcomas are wild-type P53 with variable DICER1 mutations [2]. High-grade adenosarcomas frequently harbor P53 pathway alterations [78% of cases]. Adenosarcomas with sarcomatous overgrowth had more copy number variations [2], including the amplification of 8q13 and high-level copy-number gains of MYBL1 [1]. There is no difference in the number of gene mutations between adenosarcomas with or without sarcomatous overgrowth. The most frequent mutated genes are PIK3CA/AKT/PTEN, ATRX, P53, and DICER1 [2].

## 8. NTRK-Rearranged Fibrosarcoma-like Uterine Sarcoma

Neurotrophin tropomyosin receptor kinase (NTRK) fusions are more frequently identified in mesenchymal tumors [60], thanks to the growing use of RNA-sequencing, with the first description of these neoplasms in the uterus provided in 2011 by Mills et al. [69]. These tumors were included in the 2020 WHO classification as an emerging entity identified as “NTRK-rearranged spindle cell neoplasm” and defined as a “low grade spindle cell sarcoma harboring NTRK gene rearrangements” [1]. These tumors typically affect young women (mean age 35, range 23–60) [25]. Although tumors developing in the uterine corpus and vagina have also been observed, the bulk of these neoplasms are localized in the cervix [26]. The most frequent presenting symptom is abnormal vaginal bleeding. The majority are limited to the uterus at the time of diagnosis (stage I) and the recurrence rate is up to 30% [65].

### 8.1. Pathologic Features

These neoplasms are infiltrative, with a typical “fibrosarcoma-like” morphology consisting of relatively uniform spindle cells of variable cellularity with scant cytoplasm. The cells are typically arranged in a diffuse patternless pattern, but herringbone and/or fascicular growth may also be seen. The nuclei have clumped chromatin and small nucleoli with variable mild-to-moderate atypia. The foci of the epithelioid cells or cells with cytoplasmic vacuolation can be focally present. The mitotic activity can vary from 0 to 50 per 10 high power fields [1]. Necrosis is present in some cases, and perivascular hyalinization occurs in more than half of all instances. In over 70% of cases, an inflammatory lymphoid infiltration has been documented [26]. Immunohistochemically, Pan-Trk immunohistochemistry is diffusely positive. Tumors are largely positive for S100 and CD34 [25,26] and exhibit a variable expression of SMA, but lack smooth muscle differentiation with negative desmin and h-caldesmon. PR and ER either have negative or sporadic localized weak expression. Cyclin D1 has a variable positivity. The H3K27me3 expression is retained in these tumors. BCOR is largely negative and other melanocytic markers are negative [26].

### 8.2. Molecular Landscape and Novel Therapeutic Approaches

NTRK genes code for tropomyosin receptor kinases (Trks) which comprise the NTRK1 gene (1q21.22) coding for the TrkA receptor, the NTRK2 gene (9q22.1) coding for TrkB transmembrane protein, and the NTRK3 gene (15q25) coding for TrkC glycoprotein. Once activated by the ligand binding, all of these receptors will trigger the MAPkinase, PI3 kinase/AKT pathway cascade, resulting in the activation of cell cycle, survival, and differentiation. The reported fusions in NTRK-rearranged fibrosarcoma-like uterine sarcoma include RBPMS-NTRK3, TPR-NTRK1, LMNA-NTRK1, EML4-NTRK3, and TPM3-NTRK1 gene fusions [6,28,71].

The identification of NTRK1/3 fusion transcripts has a diagnostic and therapeutic value because a targeted therapy exists [23,25,72].

## 9. SMARCA4-Deficient Uterine Sarcoma

The SWI/SNF-related, matrix-associated, actin-dependent regulator of chromatin, subfamily A, member 4 (SMARCA4)-deficient uterine sarcoma is a unique, recently discovered subtype of uterine sarcoma, with nearly 30 cases reported in the literature [2]. In terms of their clinicopathological and genetic characteristics, these tumors overlap with other SMARCA4-deficient cancers, such as ovarian small cell carcinoma of hypercalcemic type (SCCOHT), atypical teratoid/rhabdoid tumors, and thoracic sarcomas [6]. These tumors typically affect young women, with a mean age of 36 years old and clinically have a high stage at presentation [73]. This sarcoma is very aggressive and affected patients have a dismal prognosis, as most patients are not expected to live more than nine months on average [74].

### 9.1. Pathologic Features

These tumors are bulky large masses that exhibit diffuse sheets of discohesive uniform large rhabdoid and/or epithelioid characteristics with marked cytologic atypia and a high mitotic count. There is no known defining immunophenotype. EMA, CAM5.2, and CD10 may be focally positive; pan-cytokeratin, claudin 4, desmin, SMA, H-caldesmon, ER, and PR are largely negative, and the DNA mismatch repair protein expression is retained [6,73]. Unlike their ovarian counterparts, these tumors lack WT1 expression [15]. The immunohistochemistry analysis is significant for SMARCA4 (BRG) loss in the majority of cases [2,6,74]. It is important to distinguish SMARCA4-deficient uterine sarcoma from undifferentiated carcinomas, which can occasionally lose SMARCA4 expression [20]. p53 expression is wild type in SMARCA4-deficient sarcoma and mutated in undifferentiated carcinomas [2]. Furthermore, SMARCA4-deficient sarcoma shows a conservation of microsatellite stability (MSS), unlike undifferentiated carcinomas which are more often associated with microsatellite instability (MSI) [2].

### 9.2. Molecular Landscape and Novel Therapeutic Approaches

The SMARCA4-deficient sarcoma is a simple genomic sarcoma with a low mutational burden, characterized by a biallelic inactivation of the SMARCA4 gene with few other genomic alterations [2]. Germline heterozygous loss-of-function mutations in the SMARCA4 gene can result in rhabdoid tumor predisposition syndrome-2, which is associated with an increased risk of developing atypical teratoid/rhabdoid tumors and SCCOHT [73]. The identification of carriers is important because it allows for family members to be tested and appropriate screening to take place. There is no conclusive, targeted therapy for the illness yet. Targeted therapies, including PD-L1, EZH2, and CDK4/6 inhibitors, have shown promise in malignancies driven by mutations in the SWI/SNF complex, including SCCOHT [73]. Through extrapolation, these same therapies may have value in treating the SMARCA4-deficient sarcoma, so future studies are needed to explore this proposition.

## 10. COL1A1-PDGFB Fusion Uterine Sarcoma

The collagen type-1 alpha-1 (COL1A1) and platelet-derived growth factor beta chain (PDGFB) fusion uterine sarcoma is a relatively new entity that is not yet listed in the 2020 WHO classification of female genital tumors, with only a few cases reported [27]. The patients with COL1A1-PDGFB-rearranged sarcoma tend to be older than those with NTRK-rearranged sarcoma. The uterine corpus is the most commonly affected site in the female genital tract [2,28].

### 10.1. Pathologic Features

The COL1A1-PDGFB fusion uterine sarcoma has invasive borders and is highly cellular, consisting of uniform cells with ovoid-to-spindle-shaped nuclei, minimal cytoplasm, and barely perceptible cell boundaries. The cells are arranged in a storiform, herringbone architecture. Morphologically, this tumor is reminiscent of the appearance of a soft tissue dermatofibrosarcoma protuberans [28]. Mild to moderate cytologic atypia is present, and mitotic activity is often brisk (8 to 45/10 HPF) [2]. The only positive marker is CD34, although it can lose its expression if a tumor transforms into a fibrosarcoma. The smooth muscle markers, hormone receptors, and S100 are negative [28].

### 10.2. Molecular Landscape and Novel Therapeutic Approaches

The COL1A1-PDGFB fusion transcript as a result of the translocation t(17;22) (q22;q13) is linked to this sarcoma. This fusion was previously only reported in dermatofibrosarcoma protuberans in soft tissue. This molecular element may have clinical value in identifying candidates for imatinib treatment in the event of relapse who cannot be treated with debulking therapy [2,29].

## 11. FGFR1- and RET-Fusion Uterine Sarcoma

These are recently described uterine sarcomas with two case reports for each. These novel fusions were discovered during the molecular work-up of uterine mesenchymal tumors that were morphologically and/or immunophenotypically similar to the NTRK-fusion sarcoma and inflammatory myofibroblastic tumor (IMT), which expand the homology between RET- and FGFR1-fusion sarcomas and NTRK-fusion sarcomas and IMT. The two RET-fusion sarcomas occurred in young females (20 [30] and 33 [62] years old), one of them during pregnancy. Both patients eventually underwent radical hysterectomy with or without bilateral salpingectomy and peritoneal biopsies. The maximum follow-up was 24 months with no recurrence. The FGFR1-fusion uterine sarcomas were reported in older patients (48 [31] and 53 [75] years old). Both patients underwent radical hysterectomy with or without bilateral salpingectomy and peritoneal washing. The follow-up period ranged from 4 months up to 32 months. In one case, the tumor recurred at 15 months at the pelvis and lung [31]; however, both patients are still alive at the date of original report. Additional cases with clinical follow-up are needed to more accurately determine the natural history and outcomes.

### 11.1. Pathologic Features

The tumor is infiltrative and consists of a moderately cellular spindle cell proliferation arranged in a loosely fascicular-to-patternless growth pattern. The tumor cells have ovoid-to-elongated nuclei with sporadic nucleoli, and a considerable proportion of pale-to-eosinophilic cytoplasm. Scattered cytologic moderate atypia and focal coagulative tumor cell necrosis are present. The tumor can contain variable “staghorn” vasculature, with larger vessels displaying perivascular hyalinization [30,31,62,75]. The RET-fusion sarcoma has low mitotic activity, minimal lymphoplasmacytic infiltrate, and native glandular entrapment [30,62]. On the other hand, the FGFR1-fusion sarcomas show high mitotic activity in 10 HPF, moderate–diffuse lymphoplasmacytic infiltration, and native gland entrapment [31,75]. By immunohistochemistry, both sarcomas show a strong, diffuse expression of CD34 and a patchy, moderate expression of S100. The staining for cyclin D1, CD10, SMA, and PR are focal and variable. The staining for desmin, h-caldesmon, ER, melan-A, HMB45, SOX10, MiTF, myogenin, WT-1, CD117, DOG1, EMA, STAT6, and ALK were all negative. P53 staining has a wild-type nuclear expression, while H3K27me was retained [30,31,62,75].

### 11.2. Molecular Landscape and Novel Therapeutic Approaches

The rearranged during transfection (RET) gene is located on chromosome 10 and codes for a transmembrane receptor protein which belongs to the glial cell line-derived neurotrophic factor (GDNF) family ligand. Upon activation by the binding ligand, the receptor will dimerize and initiate for cell survival, migration, and differentiation. Two RET translocation partners were reported: RET-SPECC1L and TIPM3-RET [30,62]. The identification of the RET-fusion uterine sarcoma has therapeutic implications as it is one of the FDA-approved histology-agnostic targets for treatment [2]. Although only recently described in uterine sarcomas, the oncogenic role of the fibroblast growth factor receptor (FGFR) and transforming acidic coiled coil-containing protein (TACC) fusion family have been previously described in other tumors. These include glioblastoma, a small subset of “wild type” gastrointestinal stromal tumors, and extra-ventricular neurocytomas [75]. Under physiological conditions, the FGFR protein family plays key roles in cellular growth, proliferation, and survival, through the activation of downstream signaling pathways such as the MAPK and PI3K/AKT pathways [75]. The oncogenic effect of the translocation is believed to be derived from the constitutional FGFR activation through ligand-independent FGFR domine dimerization. The prognostic and therapeutic significance of the FGFR1–TACC1 fusions is uncertain, owing to their rarity. Clinical trials using small molecule FGFR inhibitors as well as broad spectrum tyrosine kinase inhibitors are ongoing [72,75].

## 12. Undifferentiated Uterine Sarcoma (UUS)

UUSs are malignant uterine mesenchymal tumors that can originate from either the endometrium or myometrium, and they lack specific lines of differentiation or immunohistochemical staining patterns [21]. They are classified as “endometrial stromal and related tumors” in the 2020 WHO classification, although they lack any histologic resemblance to proliferative-phase endometrial stroma [1]. As such, an undifferentiated uterine sarcoma is considered a diagnosis of exclusion after ruling out more common uterine mesenchymal tumors with characteristic morphologic and immunohistochemical features or recurrent chromosomal rearrangement. Due to the rarity of this tumor type and the possibility of contamination from the morphologically high-grade endometrial stromal sarcomas harboring the entity, the scope of clinicopathologic research defining the entity has been severely limited. The affected patients are postmenopausal and generally present with abnormal uterine bleeding, pelvic pain or pressure, and extrauterine symptoms. The patients diagnosed with USS have a poor prognosis with less than 2 years mean survival [21].

### 12.1. Pathologic Features

UUSs grow as large, fleshy masses that are either intramural or polypoid and have bleeding and necrosis [21]. On histologic examination, the key to the diagnostic assessment is the lack of distinct lineage differentiation both morphologically and immunophenotypically. UUSs often exhibit invasive development that is damaging to the myometrium. Undifferentiated uterine sarcomas frequently have a wide range of morphologic characteristics. Thus, it has been suggested that undifferentiated uterine sarcomas be divided into pleomorphic and uniform, based on nuclear polymorphism being minimal in the latter and marked in the former [1,21]. Immunohistochemistry is non-contributory with variable staining with b-catenin, cyclin D1, CD10, muscle markers, ER and PR [21]. Other uterine mesenchymal tumor indicators are variable in their positivity or negativity and lack well-known staining patterns.

### 12.2. Molecular Landscape

Because undifferentiated uterine sarcomas are a rare tumor type, there is a paucity of information regarding their molecular genetics. Undifferentiated uterine sarcomas frequently carry TP53 mutations and have complicated karyotypes and polysomy [1,15,21].

## 13. The Histology-Agnostic Approach: Actionable Targets among Uterine Sarcomas

A new era of drug development has begun in the last ten years, marked by histology-indifferent (agnostic), biomarker-driven therapies. This is due to the growing understanding of the molecular alterations responsible for carcinogenesis in a variety of tumor types and the accessibility of highly active, targeted therapies. Therapies are being created in this new approach to treat particular molecular abnormalities regardless of the tumor tissue of origin. As a result of the discovery of the microsatellite instability-high (MSI-H) phenotype as a predictive biomarker for the efficacy of anti-PD-1 immune-checkpoint inhibitors, histology agnostic development was first acknowledged as a novel regulatory approach for medication approvals [72]. So far, the FDA has approved six histology agnostic medications that each target a different molecular biomarker [72]. Due to their rarity and diversity, it is difficult to simultaneously investigate large numbers of sarcomas for specific targets in clinical trials and, as such, histology-agnostic treatments are of special interest in these mesenchymal tumors. In addition, the large genomic databases are of significant assistance in molecularly interrogating multiple tumors at the same time. In a recent analysis of soft tissue and bone sarcomas in the AACR GENIE database (v12.0-Public) for histology-agnostic targets, 5% of the 6955 analyzed patients could be eligible for the current FDA-approved drugs or future potential histology agnostic indications [72]. This particular study included 626 patients with uterine sarcomas with 12 different types of sarcomas, either from the primary tumors (51% of cases) or the metastasis (45% of cases). These tumors harbored either targets for approved histology agnostic medications (NTRK fusion, mismatch repair genes, BRAF V600E mutations, and RET fusion) or potential histology-agnostic targets (different mutations, gene fusions) [72]. A summary of the prevalence of these targets and type of tumors is provided in Table 2.

## 14. Conclusions

Overall, the genetic landscape of UMTs is complex and heterogeneous, with different subtypes of tumors exhibiting distinct patterns of somatic mutations, chromosomal rearrangements, and copy number alterations. The ongoing research efforts in this field promise to continue to identify new molecular signatures that can help guide clinical decision-making and improve the outcomes for patients with these rare neoplasms.

## Figures and Tables

**Table 1:** Molecular events in mesenchymal uterine tumors.

	Genetic Aberrations	References
**Leiomyosarcoma**	TP53, RB1, PTEN, and ATRX mutations	[1,3,4]
	PGR gene rearrangement	[5,6]
	PLAG1 gene fusion	[6,7]
**Low-grade endometrial stromal sarcoma**	JAZF1 and PHF1 gene rearrangements	[1,8]
**High-grade endometrial stromal sarcoma**	YWHAE rearrangements	[8,9,10,11,12,13]
	ZC3HB-BCOR rearrangements	[14,15,16,17]
	BCOR ITD	[14,15,16,17]
**Perivascular epithelioid cell neoplasms**	TSC1/TSC2 inactivation mutation	[1,18]
	TFE3, RAD51B, and HTR4-ST3GAL1 gene rearrangements	[18,19]
**Inflammatory myofibroblastic tumor**	ALK gene rearrangements (chromosome 2p23)	[1,20,21]
**Uterine tumors resembling ovarian** **sex cord tumor (UTROSCT)**	ERS1 and RHEB1 gene rearrangement	[20,22,23,24,25,26]
**Müllerian adenosarcoma**	PIK3CA/AKT/PTEN, ATRX, P53, and DICER1 mutations, 8q13 amplification, and MYBL1 copy number gain	[1,2]
**NTRK-rearranged fibrosarcoma-like** **uterine sarcoma **	RBPMS-NTRK3, TPR-NTRK1, LMNA-NTRK1, EML4-NTRK3, and TPM3-NTRK1 gene fusions	[6,27,28]
**SMARCA4-deficient uterine sarcoma**	SMARCA4 gene biallelic inactivation mutation	[20,25]
**COL1A1-PDGFB fusion uterine sarcoma**	COL1A1-PDGFB rearrangement	[20,25]
**RET-fusion uterine sarcoma**	RET-SPECC1L and TIPM3-RET rearrangements	[21,29]
**FGFR1-fusion uterine sarcoma**	FGFR1-TACC1 rearrangement	[30,31]
**Undifferentiated uterine sarcoma**	TP53 mutations, complex karyotype, and polysomy	[1,15,20]

**Table 2:** Histology-agnostic FDA-approved targets or potential targets among the cohort of uterine sarcomas in AACR GENIE v12.0-Public (n = 626 patients) [72].

Approved Histology Agnostic Targets		
Targets	Prevalence	Histologic Type
NTRK fusion	4 (0.6%)	2 undifferentiated uterine sarcomas 1 leiomyosarcoma 1 adenosarcoma
Mismatch repair gene alterations	11 (1.8%)	6 leiomyosarcoma 3 undifferentiated uterine sarcomas 1 low-grade endometrial stromal sarcoma 1 adenosarcoma
BRAF V600E mutations	1 (0.2%)	1 leiomyosarcoma
RET fusion	1 (0.2%)	1 leiomyosarcoma
Total	2.7% (17/626)	
**Potential Histology-Agnostic Targets**		
**Targets**	**Prevalence**	**Histologic Type**
KRAS G12C mutation	1 (0.2%)	Undifferentiated uterine sarcoma
KRAS G12D mutation	4 (0.6%)	Histologic type unavailable
BRCA1 mutation	1 (0.2%)	Leiomyosarcoma
BRCA2 mutation	10 (1.6%)	7 leiomyosarcomas 3 undifferentiated uterine sarcomas
ALK gene fusion	5 (1%)	2 leiomyosarcomas 1 undifferentiated uterine sarcoma 1 adenosarcoma 1 smooth muscle tumor of undetermined potential
FGFR gene fusion	2 (0.3%)	1 leiomyosarcoma 1 undifferentiated uterine sarcoma
Total	3.8% (24/626)	
